# Rural Households’ Livelihood Capital, Risk Perception, and Willingness to Purchase Earthquake Disaster Insurance: Evidence from Southwestern China

**DOI:** 10.3390/ijerph15071319

**Published:** 2018-06-23

**Authors:** Dingde Xu, Enlai Liu, Xuxi Wang, Hong Tang, Shaoquan Liu

**Affiliations:** 1 Sichuan Center for Rural Development Research, College of Management of Sichuan Agricultural University, Chengdu 611130, China; tanghongwa@126.com; 2 Geographic National Condition Monitoring Engineering Research Center of Sichuan Province, #2, Xinjun Street, Chengdu 610599, China; shandisuoliuenlai@163.com; 3 College of Land and Resources, China West Normal University, Nanchong 637000, China; wangxuxi1985@163.com; 4 Institute of Mountain Hazards and Environment, Chinese Academy of Sciences, Chengdu 610041, China

**Keywords:** livelihood capital, risk perception, disaster insurance, willingness, Wenchuan earthquake, China

## Abstract

Earthquake disaster insurance can effectively reduce the impact of earthquake disasters on rural households. Exploring rural households’ willingness to purchase earthquake disaster insurance in earthquake disaster areas provides an understanding of the motivations underlying the implementation of an insurance policy. However, few studies have examined the perspectives of rural households, in order to explore the correlations between the rural households’ livelihood capital, their disaster risk perception, and their willingness to purchase earthquake disaster insurance. A cross-sectional survey data including 241 rural households from the most severe disaster counties (cities) during the 5 • 12 Wenchuan earthquake was examined with regard to rural households’ livelihood and disaster risk perception, and ordinal logistic regression models were constructed to explore rural households’ willingness to purchase earthquake disaster insurance, as well as the driving mechanism behind this willingness. The results showed that 34.44% of rural households were very willing to purchase earthquake disaster insurance, and 7.05% of rural households were very reluctant to purchase earthquake insurance. Rural households’ livelihood capital and risk perceptions were the most important factors affecting their willingness to purchase earthquake disaster insurance. Rural households with higher scores on natural capital, physical capital, possibility, and worry were more likely to purchase earthquake disaster insurance. Specifically, keeping all other variables constant, every one unit increase in nature capital and physical capital corresponded to an increase in the odds of willingness to purchase earthquake disaster insurance by a factor of 0.14 and 0.06, respectively; every one unit increase in possibility and worry corresponded to an increase in the odds of willingness to purchase earthquake disaster insurance by a factor of 0.03 and 0.04, respectively. This study contributes to the current literature by increasing the understanding of the relationships between Chinese rural households’ livelihood capital and risk perceptions, and their willingness to purchase earthquake disaster insurance.

## 1. Introduction

China is a mountainous country; mountains cover 73.4% of the land area and are home to 45% of the population [1,2]. Mountainous areas are indispensable land space for China and can contribute to rural revitalization and economic prosperity [3,4]. However, because of the topography and geological background, China experiences one of the highest numbers of natural disasters in the world [5,6]. Earthquakes, landslides, debris flows, and other disasters are frequent in some mountainous areas; these bring great threat to residents’ lives and property safety, as well as to the wealth of farming households, which may have accumulated over decades [7,8]. According to statistics, among the 54 worst natural disasters in the world in the 20th century, eight of them have occurred in China, among which, earthquakes, floods, and typhoons have caused the greatest losses [9]. Natural disasters, led by earthquakes, have become an important factor leading rural households into poverty [2,10]. The Rural Revitalization Strategy was put forward at the 19th National Congress of the Communist Party of China (CPC). It is a general policy for “prosperous industry, livable ecology, civilized local customs, effective governance, and a wealthy life”, and defines the phase-by-phase goals of implementing the Rural Revitalization Strategy. At present, the primary task is to alleviate poverty and achieve a wealthy society by 2020 [10,11]. Since the 18th National Congress of the CPC, the alleviation of poverty in China has made remarkable progress; the impoverished rural population decreased from 98.99 million to 43.35 million, an average annual decrease of nearly 14 million. However, by the end of 2017, there were still 30 million impoverished rural people who were primarily distributed in 14 concentrated contiguous destitute areas [1,2,12,13]. These areas are mountainous and are affected by geology and terrain, including earthquakes, mudslides, and other disasters that seriously threaten people’s lives and property security. In order to achieve the central government’s poverty eradication target by 2020, and to realize rural revitalization, it is necessary to focus on the intertwined areas of poverty and disaster, with particular emphasis on the vulnerable groups in mountainous rural settlements. However, earthquakes are considered to be characterized by “small probability and great loss”. In China, a country with frequent natural disasters, it is not enough for some rural households in mountain settlements to rely only on their own recovery capabilities or on government assistance after disasters; cooperation of the government, the market, and rural households is required. In this context, catastrophe insurance, which takes into account the government, the market, and the individual’s own strength, has emerged and has become an important part of the catastrophe risk management system.

Catastrophe insurance is often considered to be an important part of residents’ preparedness for disaster prevention and for the construction of the government’s resilient disaster prevention and mitigation system [14,15,16,17,18]. As a type of catastrophe insurance, earthquake disaster insurance has attracted much attention. From a worldwide perspective, there is no lack of precedents with regard to the changes in the relevant policies of the insurance industry as a result of strong earthquakes. For instance, Japan experienced the Niigata earthquake in 1964, and in 1966, it issued the Earthquake Insurance Law, establishing the Japan Earthquake Reinsurance Co., Ltd. (Tokyo, Japan). A violent earthquake occurred in California in the United States in 1994, and the relevant laws and regulations were issued in 1995, while the California Earthquake Authority (CEA) was established in 1996. A strong earthquake occurred in Turkey in 1999, and the Turkish Catastrophe Insurance Pool (TCIP) was established in 2000. After the earthquake in Taiwan in 1999, the Measures for the Implementation of Residential Earthquake Insurance Co-insurance and Risk Commitment Mechanism was promulgated in 2001, and the Taiwan Residential Earthquake Insurance Fund (TREIF) was established in 2002 [19]. However, the construction and development of the earthquake catastrophe insurance system in mainland China has undergone a long process, which can be roughly divided into four stages. Specifically, the first stage was from 1951 to 1959. In 1951, the Government Administration Council of the Central People’s Government issued the Decision on the Implementation of Compulsory Insurance for Government Agency, State-owned Enterprise and Cooperative Properties, and Compulsory Insurance for Passengers. By the end of 1952, the vast majority of the properties of government agencies, state-owned enterprises, and cooperatives had been issued, while the insurance coverage included earthquake and other catastrophe risks. However, because of historical reasons, China’s domestic insurance business was completely suspended in 1959, and the newly established catastrophe insurance system in China came to a premature end. The second stage was from 1979 to 1994. In 1979, the State Council decided to gradually resume the domestic insurance business. The period from 1980 to 1994 was the recovery period of China’s catastrophe insurance. In this period, the scope of protection of the residents’ household property insurance included earthquake, flood, and other catastrophe risks, and the construction of the catastrophe insurance system were preliminarily improved.

The third stage was from 1995 to 2001. In 1995, from the perspective of controlling and preventing the operating risks of insurance companies, the supervision and management institutions of China’s insurance industry required insurance companies to suspend earthquake insurance. In 1996, the People’s Bank of China stipulated that flood, earthquake, typhoon, and other catastrophe risks would be eliminated from their basic responsibilities in the new enterprise property insurance clauses, which were implemented on 1 July 1996. At the same time, in 2000 and 2001, the China Insurance Regulatory Commission (CIRC) continuously issued circulars on earthquake insurance, pointing out that “earthquake insurance can only be regarded as an additional insurance for enterprise property insurance and must not be taken as the main insurance to be covered alone”. Since then, as a special additional insurance, only flood disasters were covered by various insurance companies, while devastating catastrophes such as earthquakes and tsunamis were not covered by insurance companies. The fourth stage was from 2008 to the present. In 2008, an unprecedented earthquake occurred in Wenchuan; there were nearly 70,000 deaths in Sichuan and direct economic losses of nearly 845.2 billion Yuan resulted from this devastating earthquake. This impelled researchers, policymakers, and insurance professionals to rethink the issue of the establishment of earthquake catastrophe insurance [7,20]. In 2012, an earthquake with a magnitude of 7.0 occurred in Lushan, Ya’an, Sichuan Province, leaving 196 people dead and over 10,000 people injured. This further accelerated the establishment of earthquake catastrophe insurance. In 2013, the Third Plenary Session of the 18th CPC Central Committee adopted the Decision of the Central Committee of the Communist Party of China on Some Major Issues Concerning Comprehensively Deepening the Reform, explicitly putting forward “the improvement of the economic compensation mechanism for insurance as well as the establishment of the system for catastrophe insurance”. In 2014, the pilot work of earthquake catastrophe insurance was started in Chuxiong, Yunnan, and then several cities and counties in Guangdong, Chongqing, Hebei, and Sichuan also carried out pilot work on earthquake disaster insurance. Since then, earthquake disaster insurance has been established as a system and has been incorporated into the national comprehensive disaster prevention and mitigation system.

Disaster insurance plays a positive role in transferring the impact of disasters on residents; for this reason, it has remained a hot topic in academic circles [20,21,22,23,24]. As such, residents are the participants and the major beneficiaries of disaster insurance, and their willingness to purchase disaster insurance, as well as the factors influencing this decision, have been the focus of academic research. However, in the existing studies, scholars have paid more attention to agricultural insurance [6,19,25] and flood insurance [26,27,28], whereas few studies have focused on earthquake catastrophe insurance [7,14,29]. China is a country with frequent earthquakes, and because of the objective historical reasons for the implementation of catastrophe insurance, compared with the United States, Japan, and other developed countries, the implementation of earthquake disaster insurance has only just begun in China. Thus, there are few related empirical studies of the willingness to purchase earthquake disaster insurance [19]. As a catastrophe, earthquake disasters are characterized by “small probability and great loss”; thus, residents’ willingness to purchase the corresponding insurance is different from their willingness to purchase general insurance. Generally speaking, residents prefer to insure against general risks with greater probability of occurrence, and have evasive attitudes towards catastrophe risks [26]. Therefore, for China, a country that has implemented earthquake disaster insurance for only a short period of time, the characteristics of rural households’ willingness to purchase earthquake disaster insurance in earthquake disaster threat areas requires urgent further exploration.

To date, research on the factors influencing the acquisition of disaster insurance has mostly focused on the correlations between risk perception, disaster experience, individual and family socio-economic characteristics, and willingness to purchase disaster insurance [16,17,28,30,31]. However, because of the different social, economic, political, and cultural backgrounds of the scholars, the specific mechanisms underlying the effects of these factors on residents’ insurance purchase behaviors are not uniform, and primarily depend on their respective environments [19,31,32]. For example, in terms of studies examining correlations between individual and family economic characteristics and rural households’ willingness to purchase insurance, by the authors of [28,30,31], found that family income is markedly positively correlated with residents’ purchase of disaster insurance, while the study by Jin et al. [32] reported that family income is negatively related to the residents’ purchase of disaster insurance. The latter authors suggested that a high family income among rural households mainly results from labor migration, and residents can alleviate the impact of the disaster on the family through labor migration and other methods. Therefore, they are not as willing to purchase disaster insurance. The empirical study by Wang et al. [19] showed that regional-level disaster experience is not significantly correlated with residents’ willingness to purchase disaster insurance, whereas individual-level disaster experience was clearly related to the residents’ willingness to purchase disaster insurance. However, the research by the authors of [28,32] concluded that there is a significant positive correlation between disaster experience and willingness to purchase insurance. In terms of studies examining the correlations between residents’ disaster risk perceptions/residents’ risk preferences and their purchase of insurance, by the authors of [28,30,32], found that people with risk aversion attitudes are more inclined to purchase disaster insurance. The studies by the authors of [33,34] reported that residents’ perceptions of disaster insurance are significantly positively correlated with their willingness to purchase disaster insurance. The research by the authors of [35,36,37] showed that rural households underestimate the risk of disasters, making them unwilling to purchase disaster insurance, while the empirical study by Wang et al. [19] concluded that some residents in severe disaster threat areas are unwilling to purchase insurance, and this is not because they are unaware of the threat (occurrence possibility and severity) of the disaster, but because they want the government to directly compensate them for the loss that is caused by the disaster. Therefore, in China, a country with frequent earthquake disasters, it is necessary to further explore the correlations between the respondents’ individual and family socio-economic characteristics, disaster experience, and risk perceptions, as well as their willingness to purchase earthquake disaster insurance.

Furthermore, many mountain settlements in China are areas where poverty and earthquake disasters are intertwined, as a result of the impact of disasters. In addition to the assistance from the government and society, the rural households’ own capability is also an important means to resist external shocks [10,24,38,39,40]. For example, Xu et al. [10] found that savings and migrant work income can significantly reduce the impact of external risks on rural household poverty vulnerability. Theoretically, rural households’ willingness to purchase earthquake disaster insurance is closely related to the capability of the families to resist external shocks. However, few studies have explored the correlations between rural households’ capability and their willingness to purchase earthquake disaster insurance. In existing studies, scholars have mostly characterized the strength of rural households’ capability in accordance with the amount of their livelihood capital [2,41,42,43]. Therefore, the correlations between rural households’ livelihood capital and their willingness to purchase earthquake disaster insurance require further exploration.

Sichuan is a typical area in China where geological disasters and poverty are intertwined. Eight earthquake belts cross the four major poverty areas in Sichuan Province (Plateau Tibet area, Daliang Mountain and Xiaoliang Mountain Yi area, Qinba Mountain area, and Wumeng Mountain area; these are also the concentrated distribution areas of the 45 national poverty counties in Sichuan Province). For example, the Longmenshan earthquake belt crosses Wenchuan County and Lushan County, and these two counties successively experienced the Wenchuan earthquake and the Lushan earthquake. Since the 18th National Congress of the Communist Party of China, Sichuan has made outstanding achievements in the alleviation of poverty, and the poor rural population has decreased from 7.5 million to 1.71 million. However, the remaining poor population is primarily concentrated in the four major poverty areas, where there are frequent earthquake disasters; this makes it difficult to achieve rural revitalization and economic prosperity for the whole society. In the past 10 years, earthquake disasters have occurred frequently in Sichuan, resulting in considerable losses. The 5 • 12 Wenchuan earthquake, the 4 • 20 Lushan earthquake, and the 8 • 8 Jiuzhai Valley earthquake are world-famous. Among them, the most destructive earthquake since the founding of the People’s Republic of China, the Wenchuan earthquake, was the most typical earthquake, the direct economic losses reached 845.215 billion Yuan. At the same time, there are numerous mudslide and landslide disaster points around the earthquake belts, and unlike the low frequency of earthquake disasters, these geological disasters occur frequently, which is another factor contributing to the poverty of rural households in mountain settlements. In areas at high risk of earthquake disasters, it is crucial to establish a resilient disaster prevention system, and earthquake disaster insurance is one of the core aspects of the construction of a regional resilient disaster prevention system.

Based on this, this study explores the relationships between household livelihood capital, risk perception, and willingness to purchase earthquake disaster insurance among farming households. The findings of this study will inform disaster prevention policies. Generally, this study attempted to answer the following two questions:

(1) What are the characteristics of willingness to purchase insurance among rural households in the Wenchuan earthquake severe disaster areas?

(2) What are the specific mechanisms of the relationships between household livelihood capital, risk perception, and willing to purchase earthquake disaster insurance among rural households?

Based on the literature, and with consideration to the actual situation in the research area (the Wenchuan earthquake zone), the following three hypotheses were proposed:

**Hypothesis 1** **(H1).** The five types of livelihood capital of rural households will be significantly related to the willingness to purchase earthquake disaster insurance; the higher the score for the five types of livelihood capital, the stronger the willingness to purchase earthquake disaster insurance among rural households.

**Hypothesis 2** **(H2).** Risk perception will be significantly related to willingness to purchase earthquake disaster insurance. Respondents with a higher perception of possibility and worry will be more likely to purchase earthquake disaster insurance, while respondents with a higher perception of controllability will be less likely to purchase earthquake disaster insurance.

**Hypothesis 3** **(H3).** Respondents’ personal characteristics and household characteristics will be significantly related to their willingness to purchase earthquake disaster insurance; however, the directions of these relationships are unknown.

## 2. Data and Methods

The 5 • 12 Wenchuan earthquake occurred on 12 May 2008 (Beijing time) at 14:28:04. The surface wave magnitude, the moment magnitude, and the earthquake intensity reached 8.0 Ms, 8.3 Mw, and 11 degrees, respectively. The earthquake affected more than half of China, as well as many other Asian countries and regions. The tremor was felt from Liaoning in the north, to Shanghai in the east and to Hong Kong, Macao, Thailand, Vietnam, and Pakistan in the west. Within China’s geographical area, the severe earthquake disaster area covered an area of more than 100,000 km^2^. In this area, there were 10 most severe disaster counties (cities), 41 severe disaster counties (cities), and 186 general disaster counties (cities). As of 12:00 on 18 September 2008, the 5 • 12 Wenchuan earthquake led to a total of 69,227 deaths, 374,643 injured persons, and 17,923 missing persons, becoming the most destructive earthquake since the founding of the People’s Republic of China, and the most deadly earthquake after the Tangshan earthquake.

### 2.1. Data Source

The data for this study mainly came from a questionnaire survey conducted in September 2015 in the Wenchuan earthquake’s most severe disaster areas. The investigation largely focused on the situation of rural households in 2014 in terms of the livelihood capital, disaster risk perception, and behavioral strategies to avoid disaster. Each questionnaire took approximately half an hour to complete. This study selected sample counties (cities) from the 10 most severe disaster counties (cities) during the Wenchuan earthquake, using stratified random sampling.

**Figure 1:**
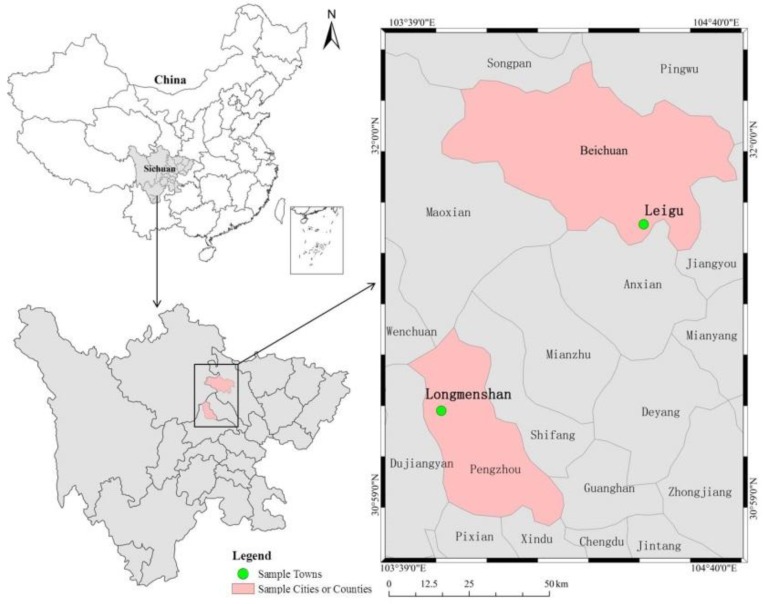
Locations of sample counties (cities) and sample towns.

Specifically, according to the differences in economic development levels, the 10 most severe disaster counties (cities), namely Wenchuan County and Mao County in Aba Prefecture, Beichuan County, An County and Pingwu County in Mianyang, Mianzhu City and Shifang City in Deyang, Qingchuan County in Guangyuan, and Dujiangyan City and Pengzhou City in Chengdu, were randomly divided into two groups, and one county (city) was randomly selected from each group to represent the sample county (city). Beichuan County (representing a low economic development level, with a per capita GDP of 17,052 Yuan in 2013, and 162,047 people were affected by the Wenchuan earthquake) and Pengzhou City (representing a high economic development level, with a per capita GDP of 30,736 Yuan in 2013, and 400,000 people were affected by the Wenchuan earthquake) were selected as the sample counties. Next, taking full account the disaster severity of the sample counties (cities), together with the differences in economic and social development levels and ethnic distribution, this study selected Leigu Town in Beichuan Qiang Autonomous County and Longmenshan Town in Pengzhou City as the sample towns (according to statistics, the two sample towns were seriously affected by the Wenchuan earthquake in 2008, and the collapse rates of houses in these two towns were both over 75%) (Figure 1). Among them, Leigu Town (including 30 villages and 18,229 people who were affected by the Wenchuan earthquake) is located in the southeast of Beichuan County, and the Han people and Qiang ethnic groups live together in this territory. The terrain gradually decreases from west to east, and there is a complex geological environment and frequent mountain disasters. Leigu Town is about 60 km away from downtown Mianyang. However, Longmenshan Town (including 6 villages and 14,501 people who were affected by the Wenchuan earthquake) is located in the northern mountainous area of Pengzhou and in the transition zone of the Longmenshan fault zone. This town has numerous mountains, great altitude differences, abundant tourism resources, a high economic development level, and is 38 km away from downtown Pengzhou. Finally, according to the differences in the economic development level of villages, 4–5 villages were randomly selected from the towns to act as sample villages, and 20–36 rural households were randomly selected from these towns to act as the sample rural households to participate in the final investigation. On average, there were about four people living e in each household. In the course of the investigation, investigators with enumeration and sampling skills, and who were well versed in the study context, were involved in data collection. After data entry, the results were collated and summarized. A total of 248 questionnaires were obtained from nine sample villages in two sample towns, and after the invalid questionnaires were excluded, a total of 241 valid questionnaires (124 from Beichuan and 117 from Pengzhou) were obtained; thus, the valid questionnaire rate was 97.17% (Table 1).

### 2.2. Methods

#### Selection and Definition of Model Variables

The aim of this study was to explore the driving mechanism behind rural households’ willingness to purchase earthquake disaster insurance from the perspective of rural households’ livelihoods and disaster risk perceptions. To achieve this goal, several key variables were measured. The key variables are described below.

(1) Dependent variable

The dependent variable was rural households’ willingness to purchase earthquake disaster insurance, which was measured on a Likert scale ranging from 1–5 (1 = strongly unwilling, 2 = unwilling, 3 = neutral, 4 = willing, and 5 = strong willing).

(2) Independent (focus) variables

The core variables of this study were rural households’ livelihood capital and disaster risk perceptions.

(a) Livelihood capital measurement

The measurement of rural households’ livelihood capital was based on the sustainable livelihoods analysis framework from the Department for International Development (DFID); the livelihood capital was divided into human capital, natural capital, social capital, financial capital, and physical capital. With consideration to the research of the authors of [1,2,8,10,42,43,44,45,46,47], together with the actual situation in the study area, the above five types of livelihood capital were measured with different detailed variables (Table 2). Finally, consistent with the research of Peng et al. [8], the entropy method was used to deal with the variables in Table 2, and finally, an objective value for the five types of livelihood capital of rural households was obtained (for a detailed introduction to the entropy method please see Peng et al. [8] and Appendix A).

(b) Risk perception measurement

The current study adopted the psychological measurement paradigm of Slovic [47], which suggests that disaster risk perception is a multi-dimensional concept. With consideration to the actual research area of China, and with reference to the measurement of risk perception in the studies by the authors of [10,48,49,50,51,52,53,54,55,56], this study measured risk perception with respect to the following three dimensions, namely, possibility, worry, and controllability (Table 3). Before the factor analysis, Cronbach’s alpha reliability tests were carried out for each measure of risk perception. The results showed that the Cronbach’s alphas for the scales possibility, worry, and controllability were 0.72, 0.80, and 0.83, respectively; these values are within the acceptable range and indicate that these measures are suitable for subsequent analysis. Next, a factor analysis was performed for the dimension reduction analysis (the KMO test value of the factor analysis was 0.643, indicating that the data is suitable for factor analysis) so as to obtain a comprehensive score for the three dimensions of possibility, worry, and controllability. The results of the factor analysis showed that each of the variables loaded exclusively on its hypothesized factor, and the cumulative variance contribution rate of the model was 68.56%. Finally, consistent with the study of Xu et al. [11], the efficacy coefficient method was used to convert the above three dimension scores into percentage scores (for the introduction of efficacy coefficient method, please see Appendix B).

(3) Control variables

In order to test the robustness of the focus variables, several control variables that reflect the characteristics of the individual respondents (e.g., age, gender, number of years of education, etc.) and the rural households (e.g., source of information, housing structure, etc.) were added to the model. For information on the definition and measurement of variables please see Table 4.

### 2.3. The Models

The dependent variable in this study was an ordered multi-classification variable (willingness to purchase earthquake disaster insurance among rural households), and the independent variables included category variables and continuous variables. Therefore, an ordinal logistic regression model was constructed to explore rural households’ willingness to purchase earthquake disaster insurance and the driving mechanisms underlying this willingness. The formula is as follows:

(1) $$\;Logit(Y_{i})=\beta_{0i}+\beta_{1i}*LC_{i}+\beta_{2i}*RP_{i}+\beta_{3i}*Con_{i}+\varepsilon_{i}$$

In Formula (1), $\;Y_{i}$ represents the dependent variable of the model; $LC_{i}$ and $RP_{i}$ indicate the focus variables of the model, namely the indicators representing household livelihood capital and risk perception, respectively; $Con_{i}$ denotes the control variable of the model; $\beta_{0i}$, $\;\beta_{1i}$, $\;\beta_{2i}$, and $\beta_{3i}$ refer to the parameters to be estimated for the model; and $\varepsilon_{i}$ is the residual of the model. The software used was Stata 15.0 (StataCorp. LLC, College Station, TX, USA).

## 3. Results

### 3.1. Descriptive Statistics

Table 4 shows the definitions and descriptive statistics for the variables in the model. For the dependent variable, 34.44% (83 households) of respondents were very willing to purchase earthquake disaster insurance, while 7.05% (17 households) were very reluctant to purchase earthquake insurance. For the independent variables of focus, the rural households’ human capital and physical capital scores were relatively high, with an average of 13.42 points and 10.75 points, respectively; the rural households’ financial capital, social capital, and natural capital scores were relatively low, with averages of 3.34 points, 3.27 points, and 1.24 points, respectively. Interestingly, the average score for the three dimensions of disaster risk perception was 60 points, with little variation (the standard deviation of the maximum possible dimension was only 8.65 points). With regard to the control variables, 43% of the respondents were male, the average age of all of the respondents was 54.56 years, and the average educational level was 4.99 years; and 25% of the respondents had experienced landslides. In terms of housing structure, the proportion of houses made of bricks and tiles was 54.77% (132 households), while 8.71% (21 households) were civil structure houses that were mainly made of mud and wood, and the rest were reinforced concrete structures, accounting for 36.51% (88 households). With regard to access to information channels, the majority of respondents accessed media channels for earthquake disaster information (51.45%), followed by official channels (40.66%), and private information (only 19 households).

### 3.2. Psychometric Model Results

Table 5 shows the correlation coefficient matrix of model variables. As shown in Table 5, most correlations of the variables were below 0.3 and only a few were above that cut point (for example, the correlation between human capital and age, human capital and education, housing material and information, etc.), indicating that there was no serious multicollinearity problem between variables. Meanwhile, before constructing the models, we tested whether there was serious multicollinearity between the independent variables. The results showed that the variance inflation factor was less than 10, indicating no evidence of multicollinearity. Table 6 shows the regression estimates for the analysis of households’ willingness to purchase earthquake disaster insurance. In order to test the robustness of our focus variables, five regression models were constructed. Model 1 and model 2 include only rural households’ livelihood capital and risk perceptions, respectively, model 3 includes both rural households’ livelihood capital and risk perceptions, while model 4 and model 5 includes all of the independent variables (that is, focus variables and control variables). Among them, in model 1–model 4, the odds ratio is reported in the regression results, while in model 5, the correlation coefficients are reported in the regression results. Additionally, in order to eliminate the influence of heteroscedasticity on the model results, all of the models used robust standard errors. According to the ward test statistics, all of the models were significant overall at the 0.1 level (*p* < 0.1), indicating that at least one independent variable was significantly correlated with the dependent variables in each model. In subsequent analyses, we focused only on the significance of the variables in the models.

As can be seen from model 1, model 3, and model 4, consistent with part of research hypothesis H1, only the natural capital and physical capital were positively related with willingness to purchase earthquake disaster insurance; the results were robust. Based on this, model 4 and model 5 were primarily used to explain the results. Although the correlations between human capital, social capital, financial capital, and rural households’ willingness to purchase earthquake disaster insurance were positive, they were not significant. Specifically, keeping all of the other variables constant, every one unit increase in nature capital and physical capital corresponded to an increase in the odds of willingness to purchase earthquake disaster insurance by a factor of 0.14 and 0.06, respectively.

Similarly, consistent with part of research hypothesis H2, risk perception was an important factor affecting households’ willingness to purchase earthquake disaster insurance. Possibility and Worry were positively related with willingness to purchase earthquake disaster insurance, and the results were robust. The correlation between Controllability and willingness to purchase earthquake disaster insurance among rural households was not significant. Specifically, keeping all other variables constant, every one unit increase in possibility and worry corresponded to an increase in the odds of willingness to purchase earthquake disaster by a factor of 0.03 and 0.04, respectively. Interestingly, inconsistent with H3, regardless of the model, the relationships between the respondents’ personal characteristics and household characteristics, and the household willingness to purchase earthquake disaster insurance were not significant.

## 4. Discussion

China is one of the most important mountain countries in Asia and has a very important position in the world. However, China is also one of the most earthquake-prone countries in the world. The extreme environment after the earthquake may lead to an increase in the prevalence of various infectious diseases, and seriously threaten the residents’ health in the disaster areas. In order to construct a sustainable mountain society, to help the residents of the disaster areas rationally respond to extreme environmental conditions, it is of great importance to explore the protection mechanism of catastrophe insurance and its influencing factors. Based on this, this study used survey data from the Wenchuan earthquake disaster area to analyze the characteristics of rural households’ willingness to purchase earthquake insurance, and to explore the relationships between livelihood capital, risk perception, and willingness of rural households to purchase earthquake disaster insurance. Compared with the existing research, the marginal contribution of this study was that econometric models were used to reveal the mechanisms underlying the willingness to purchase earthquake disaster insurance from the perspective of rural households’ abilities and risk perceptions. There were some similarities and differences between the current research results and the existing studies.

This study found that livelihood capital is an important factor that influences the willingness to purchase earthquake disaster insurance, and that the amount of livelihood capital is directly related to the strength of the rural households’ response to the earthquake shocks; rural households’ with a strong resilience can recover quickly from the earthquake disaster, physiologically (for example, household members can be comforted by the fact that they are injured by the earthquake, can use the capital to recover quickly) and psychologically (rural households have more capital to use for post-disaster recovery, which can reduce the psychological burden), and all get more relief. Specifically, there was a significant correlation between natural capital and physical capital of rural households and their willingness to purchase earthquake disaster insurance. However, the correlations between human capital, social capital, and financial capital, and willingness to purchase earthquake disaster insurance were not significant. This is an interesting finding. For rural households, physical capital is a symbol of family wealth. To improve quality of life, contribute to their children’s marriages, and because of other considerations, rural households need to convert many years of family income to family houses or other consumer durables. Hence, physical capital is the property that rural households want to specially protect when an earthquake occurs; if this kind of capital is more abundant, the household will be more willing to purchase earthquake disaster insurance. Land is the ultimate guarantee for the livelihood of rural households, and many rural households who do not go out to work depend on the land for living; because of the long-term dual division of urban and rural areas in China, many migrant workers are unable to enjoy the welfare of urban residents, so even rural households that go out to work will generally choose to go back home and rely on the land for living when they are old. Therefore, land capital is immovable, but it is a livelihood asset that rural households are particularly worried about when an earthquake disaster occurs. On the other hand, compared with physical capital and natural capital, human capital, social capital, and financial capital are movable, and even if an earthquake occurs, rural households do not need to worry too much about these capitals. This may be why they were not significantly related with willingness to purchase earthquake disaster insurance. Furthermore, the current study focused on how poverty and earthquake disasters are intertwined. Rural households’ human capital and financial capital are not high, while their social capital is highly homogenous, and the insufficiency of rural households’ own capability can also lead to their lack of willingness to purchase earthquake disaster insurance. In addition, when an earthquake occurs, which is considered to be a catastrophe characterized by “small probability and great loss”, it is generally not sufficient for rural households to rely only on their own capability to cope with the impact of the disaster, rather, they tend to expect the government to cover major disaster losses.

There are similarities and differences between the current results and those by the authors of [19,28,30,32,33,34], who found that the respondents’ risk perceptions are also an important factor that affect the households’ willingness to purchase earthquake disaster insurance. In this study, consistent with research hypothesis H2, the higher the scores for possibility and worry, the greater the willingness of rural households to purchase earthquake disaster insurance. However, inconsistent with research hypothesis H2, the correlation between controllability and willingness to purchase earthquake disaster insurance among rural households was not significant. This may be because rural respondents view earthquakes as catastrophic and devastating, and the serious casualties caused by the Wenchuan earthquake in 2008 may have left a lasting impression on rural households; after this experience, rural households may believe that it is difficult to prevent and control earthquakes, and thus, their perception of the controllability of earthquakes was not significantly related with their willingness to purchase earthquake disaster insurance.

In this study, the correlations between the control variables and willingness of rural households’ to purchase earthquake disaster insurance were not significant. This is consistent with research [57,58], as Lindell et al. reported, the evidence for these variables as predictors of actual or expected insurance purchase is mixed, different studies have different results, so the nonsignificant findings in this study are not particularly surprising.

Although this study can provide a novel contribution to the existing research, there are still some deficiencies. For example, this study only focuses on rural households’ willingness to purchase earthquake disaster insurance and the factors influencing this willingness among the residents of the most severe disaster areas during the Wenchuan earthquake; thus, it is unknown whether the research results are applicable to the severe disaster areas and the general disaster areas. Furthermore, compared with landslides, mudslides, and other mountain disasters, earthquake disasters are characterized by low frequency and high destruction. Therefore, it is also unknown whether the results and conclusions of this study are generalizable to rural households in settlements at risk of landslide, mudslide, and other mountain disasters. In future research, the scope of the research can be further expanded to explore the relationships between rural households’ livelihood capital and disaster risk perception, and their willingness to purchase disaster insurance in settlements at risk of different types of disasters and with different degrees of threat risk. Meanwhile, this study does not explore the amount of money that rural households are willing to pay for insurance, which could also be further studied in the future. Moreover, similar to most studies, this study also uses *p* < 0.1, *p* < 0.05, and *p* < 0.01 as the criteria to determine the significance of variables. In fact, in some empirical studies of psychology, in order to avoid the influence of the excessive experiment-wise error rate on the model results, scholars often use *p* < 0.05 and *p* < 0.01 as the criterion to judge the significance of variables. By this standard, Nature capital in this study will become non-significant. Meanwhile, several other significant variables in the models will also become non-robust. Therefore, in future studies, the criterion of significance of variables can be further tightened.

## 5. Conclusions

Using survey data from the most severe disaster counties (cities) during the 5 • 12 Wenchuan earthquake, ordinal logistic regression models were constructed to explore the influence of livelihood capital and disaster risk perception on rural households’ willingness to purchase earthquake disaster insurance. The results showed the following:

(1) Rural households’ livelihood capitals are important factors affecting rural households’ willingness to purchase earthquake disaster insurance. Rural households with higher scores on natural capital and physical capital were more likely to purchase earthquake disaster insurance. When keeping all other variables constant, every one unit increase in natural capital and physical capital corresponded to an increase in the odds of willingness to purchase earthquake disaster insurance by a factor of 0.14 and 0.06, respectively.

(2) Respondents’ risk perception is also an important factor that affects households’ willingness to purchase earthquake disaster insurance. Rural households with higher scores on possibility and worry were more likely to purchase earthquake disaster insurance. When keeping all other variables constant, every one unit increase in possibility and worry corresponded to an increase in the odds of willingness to purchase earthquake disaster insurance by a factor of 0.03 and 0.04, respectively.

(3) Interestingly, regardless of the model, the relationships between respondents’ personal characteristics, household characteristics, and household willingness to purchase earthquake disaster insurance were not significant.

The results of this study also have important policy implications. For example, the study found a positive correlation between livelihood capital, disaster risk perception, and willingness to purchase earthquake disaster insurance. This suggests that, when implementing earthquake disaster insurance, the government should increase the amount of insurance compensation for earthquake disasters in a moderate way, thus providing policyholders with the funds they need to replace the property that is damaged or destroyed and enhancing rural households’ perception of disasters, so as to reduce barriers to the implementation of earthquake disaster insurance in severe earthquake disaster areas and to effectively reduce property loss of rural households.

## Figures and Tables

**Table 1:** Summary statistics for sample farming households in sample villages and counties.

Sample County/City and Sample Town	Sample Village	Sample Households	Total
Beichan County Leigu Town	Gaitou Village	35	124
	Longtou Village	25	
	Pingshang Village	20	
	Tianba Village	24	
	Tianping Village	20	
Pengzhou City Longmenshan Town	Jiufeng Village	36	117
	Tuanshan Village	35	
	Sangou Village	26	
	Guoping Village	20	
Total	241		

**Table 2:** The measurement index system for measuring livelihood capital of farming households.

Capital Type	Variable	Variable Description and Definition	Mean	SD ^a^
Human capital	Hedu	Years of education of household head (years)	5.39	3.61
	Lab	Number of laborers in farming household (number)	2.74	1.55
Nature capital	Land	Farming households cultivated land area (mu ^b^)	3.10	5.04
Social capital	Cash	The annual amount of gift money (Yuan ^c^)	3107.88	3474.66
	Cadre	Whether any of the relatives of the farming households are village cadres? (0 = no, 1 = yes)	0.10	0.32
Financial capital	Save	Total savings of farming households (Yuan)	5548.96	1812.41
	Income	Total annual cash income of farming households (Yuan)	2490.95	2265.65
Physical capital	Goods	Number of durable consumer goods in farming household (number)	3.25	1.21
	House	Living space per person (m^2^/person)	36.22	27.57

Note: ^a^ SD—standard deviation; ^b^ 1 mu ≈ 667 m^2^ or 0.667 ha; ^c^ 1 USD = 6.19 Yuan (at the time of the study).

**Table 3:** Risk perception measurement.

Entry Code	Dimension	Item ^a^	Mean	SD ^b^
A1	Possibility	I always feel that an earthquake will come one day (1–5).	3.12	1.32
A2		We have a greater risk of earthquakes than any other regions (1–5).	3.51	1.17
A3		I think the risk of earthquake disaster is increasing here in recent years (1–5).	3.19	1.19
A4		In the next 10 years, there will be earthquakes near my home (1–5).	3.05	1.11
A5	Worry	When I think of an earthquake, I feel afraid (1–5).	4.42	1.12
A6		I’m worried about the impact of an earthquake on the village and the family (1–5).	4.40	1.03
A7		In the event of a disaster, I think the sky is falling (1–5).	3.98	1.21
A8	Controllability	Although earthquakes are not controllable, there are some measures I can take (such as strengthening the house) to reduce the loss (1–5).	4.20	0.90
A9		There are reasonable ways, such as governance, to reduce other disasters caused by earthquakes (1–5).	4.20	0.75

^a^ 1—totally agree, 2—agree, 3—neutral, 4—disagree, and 5—totally disagree; ^b^ SD—standard deviation.

**Table 4:** Definition and descriptive statistics of the variables in the model.

Category	Variable	Measure	Mean	SD
Dependent variable	Insurance	Willingness to purchase earthquake disaster insurance (1 = strongly unwilling, 2 = unwilling, 3 = neutral, 4 = willing, and 5 = strong willing)	3.65	1.23
Focus independent variable	Human	Scores for human capital of farming households (1–100)	13.42	6.47
	Nature	Scores for nature capital of farming households (1–100)	1.24	2.02
	Social	Scores for social capital of farming households (1–100)	3.27	5.86
	Financial	Scores for financial capital of farming households (1–100)	3.34	3.56
	Physical	Scores for physical capital of farming households (1–100)	10.75	3.61
	Possibility	Scores for perception of the possibility of an earthquake (1–100)	60.00	8.65
	Worry	Scores for worry about an earthquake (1–100)	60.00	8.59
	Controllability	Scores for perception of controllability in an earthquake (1–100)	60.00	6.79
Control independent variable	Gender	Responder gender (0 = female, 1 = male)	0.43	0.50
	Age	Responder age (years)	54.56	14.06
	Education	Years of education (year)	4.99	3.69
	Nationality	Responder nationality (0 = Qiang, 1 = Han)	0.79	0.41
	Experience	Whether an earthquake has been experienced (0 = no, 1 = yes)	0.25	0.44
	Structure	Housing material (1 = civil, 2 = tile, 3 = concrete)	2.28	0.61
	Information	Information channel (0 = private, 1 = official, 2 = media)	1.44	0.64

**Table 5:** Pearson product moment correlation coefficient or Spearman rank order correlation matrix of model variables.

	1	2	3	4	3	4	5	6	7	8	9	10	11	12	13	14	15	16	17	18
1 insurance	1																			
2 human	0.07	1																		
3 nature	0.13 **	0.14 **	1																	
4 social	0.08	0.18 ***	0.16 **	1																
5 financial	0.09	0.33 ***	0.13 **	0.38 ***	1															
6 physical	0.18 ***	0.23 ***	0.09	0.17 ***	0.25 ***	1														
7 possibility	0.16 **	0.10	0.10	−0.06	−0.00	0.12 *	1													
8 worry	0.13 **	−0.03	0.07	0.04	0.07	0.05	0.00	1												
9 controllability	0.04	0.10	−0.08	−0.07	−0.04	−0.09	−0.00	0.00	1											
10 gender	−0.08	0.09	0.12 *	0.06	0.18 ***	0.03	−0.12 *	0.07	−0.27 ***	1										
11 age	−0.12 *	−0.48 ***	−0.05	−0.05	−0.10	−0.16 **	−0.05	−0.02	−0.05	0.02	1									
12 education	0.08	0.54 ***	0.03	0.17 ***	0.25 ***	0.28 ***	−0.01	0.04	−0.16 **	0.28 ***	−0.56 ***	1								
13 nationality	−0.06	−0.11 *	−0.08	0.02	−0.07	−0.17 ***	−0.22 ***	0.06	−0.06	0.12 *	0.13 **	0.07	1							
14 experience	−0.06	0.14 **	−0.08	0.04	0.08	−0.06	0.01	−0.07	0.03	0.11 *	−0.20 ***	0.20 ***	−0.00	1						
15 infor1	−0.04	−0.22 ***	−0.02	−0.12 *	−0.13 **	−0.09	−0.06	0.02	−0.03	−0.03	0.11 *	−0.15 **	0.04	−0.03	1					
16 infor2	0.02	0.00	−0.03	0.03	0.13 **	0.09	0.06	−0.13 **	0.07	0.02	0.12 *	−0.08	−0.05	−0.02	−0.24 ***	1				
17 infor3	0.00	0.12 *	0.04	0.04	−0.06	−0.04	−0.03	0.12 *	−0.05	0.00	−0.18 ***	0.16 **	0.03	0.03	−0.30 ***	−0.85 ***	1			
18 struc1	0.00	0.05	0.07	0.04	−0.02	−0.01	0.01	0.02	−0.04	0.15 **	0.06	−0.05	−0.02	0.02	−0.04	0.01	0.01	1		
19 struc2	0.10	0.22 ***	0.26 ***	0.06	0.08	0.15 **	0.18 ***	−0.10	0.04	0.03	−0.28 ***	0.08	−0.37 ***	−0.05	−0.11	0.02	0.03	−0.34 ***	1	
20 struc3	−0.11 *	−0.26 ***	−0.31 ***	−0.08	−0.07	−0.15 **	−0.19 ***	0.09	−0.02	−0.12 *	0.25 ***	−0.05	0.39 ***	0.03	0.13 **	−0.03	−0.04	−0.23 ***	−0.83 ***	1

Note: Lower-triangular cells report Pearson’s correlation coefficients, upper-triangular cells are Spearman’s rank correlation; ***, **, and * refers to *p* < 0.01, *p* < 0.05, and *p* < 0.1, respectively; infor1, infor2, and infor3 refer to information from private, official, and media, respectively, while struc1, struc2, and struc3 refer to housing material is civil, tile, or concrete, respectively.

**Table 6:** Results of regression estimates of households’ willingness to purchase earthquake disaster insurance ^a^.

Variables	Model 1	Model 2	Model 3	Model 4	Model 5
Human	0.01		0.00	−0.02	−0.02
	(0.02)		(0.02)	(0.03)	(0.03)
Nature	0.15 *		0.13 *	0.14 *	0.14 *
	(0.08)		(0.07)	(0.08)	(0.08)
Social	0.00		0.01	0.01	0.01
	(0.02)		(0.02)	(0.02)	(0.02)
Financial	0.01		0.01	0.02	0.02
	(0.03)		(0.03)	(0.04)	(0.04)
Physical	0.08 **		0.07 **	0.06 *	0.06 *
	(0.03)		(0.03)	(0.03)	(0.03)
Possibility		0.04 **	0.03 **	0.03 *	0.03 *
		(0.02)	(0.02)	(0.02)	(0.02)
Worry		0.04 **	0.04 **	0.04 *	0.04 *
		(0.02)	(0.02)	(0.02)	(0.02)
Controllability		0.01	0.02	0.01	0.01
		(0.01)	(0.01)	(0.02)	(0.02)
Gender				−0.37	−0.37
				(0.28)	(0.28)
Age				−0.01	−0.01
				(0.01)	(0.01)
Education				0.03	0.03
				(0.05)	(0.05)
Nationality				0.17	0.17
				(0.34)	(0.34)
Experience				−0.20	−0.20
				(0.30)	(0.30)
Structure = 2 ^b^				−0.20	−0.20
				(0.55)	(0.55)
Structure = 3 ^b^				−0.33	−0.33
				(0.55)	(0.55)
Information = 1 ^c^				0.19	0.19
				(0.46)	(0.46)
Information = 2 ^c^				0.12	0.12
				(0.43)	(0.43)
Observations	241	241	241	241	241
Wald chi2 (χ^2^)	11.02	11.60	22.89	24.94	24.94
Prob > chi2 (χ^2^)	0.05	0.01	0.00	0.09	0.09
Pseudo R^2^	0.02	0.02	0.03	0.04	0.04

Note: ^a^ The values inside the parentheses are robust standard errors, while the values outside the parentheses are odds ratio (in model 1–model 4) or correlation coefficients (in model 5); *, **, and *** refer to *p* < 0.1, *p* < 0.05, and *p* < 0.01, respectively; ^b^ The reference category is Structure = 1 (civil); ^c^ The reference category is Information = 0 (private).

## Appendix A. The Entropy Method Steps for Measuring Rural Households’ Livelihood Capital

### (1) Dimensionless processing of index data

Because the original index has different dimensions, it is necessary to conduct dimensionless processing for comparison purposes. The process can be expressed by Formula (A1), as follows:

(A1) $$Y_{ij}=\frac{X_{ij}-\min(X_{1j},X_{2j},\cdots ,X_{nj})}{\max(X_{1j},X_{2j},\cdots ,X_{nj})-\min(X_{1j},X_{2j},\cdots ,X_{nj})}*100,\;i=1,2,\cdots ,n;\;j=1,2,\cdots ,m$$

where $Y_{ij}$ indicates the score of the original index data after dimensionless processing; $X_{ij}$ indicates the actual value of the sample; $X_{max}$ indicates the maximum value of the index series; and $X_{min}$ indicates the minimum value of the index series.

### (2) Calculation of proportion of index values

$P_{ij}$, the proportion of the i rural household index value of the j index, can be calculated by Formula (A2), as follows:

(A2) $$P_{ij}=Y_{ij}/\sum_{1}^{m}Y_{ij},\;m=1,2,3\cdots$$

### (3) Calculation of entropy value of index

$E_{j}$, the entropy value of the index, can be calculated by Formula (A3), as follows:

(A3) $$E_{j}=-k\sum_{i=1}^{m}P_{ij}\ln(P_{ij})$$

where *k* > 0, ln is the natural logarithm. For example, if  k=1/ln(m), then 0 ≤ *E_j_* ≤ 1.

### (4) Calculation of difference coefficient of index

$G_{j}$, the difference coefficient of the j index, can be expressed by Formula (A4), as follows:

(A4) $$G_{j}=1-E_{j}$$

where $G_{j}$ reflects the differences of the value of the index data. If the difference of the data is greater, both $G_{j}$ and the weight of the index will be greater; when the data of a certain index are equal, the difference coefficient is the smallest, reaching 0.

### (5) Determination of index weight

$W_{j}$, the index weight, can be determined by Formula (A5), as follows:

(A5) $$W_{j}=G_{j}/\sum_{j=1}^{n}G_{j},\;n=1,2,3\cdots$$

### (6) The calculation of the evaluation score of the single index of rural household

$S_{ij}$, the evaluation score of the j index of rural household i, can be calculated by Formula (A6), as follows:

(A6) $$S_{ij}=W_{j}*Y_{ij}$$

### (7) Calculation of types of rural households’ livelihood capital score

After the weight and evaluation score of each index are determined, the rural households’ livelihood capital score can be obtained by adding up the composite scores of the various indexes in each dimension. Finally, we got $HC_{i}$, $\;NC_{i}$, $\;FC_{i}$, $\;SC_{i}$, $\;PC_{i}$, $\;CP_{i}$, which respectively indicate rural households’ human capital, natural capital, financial capital, social capital, and physical capital.

## Appendix B. The Introduction of Efficacy Coefficient Method

The efficacy coefficient method is also called the efficacy function method and it is according to the principle of multi-objective programming; for each evaluation index, a satisfactory value and an unallowable value are determined, the maximum value is the upper limit, and the unallowable value is the lower limit. The degree of satisfaction of each index is calculated, and the score of each index is determined, then, the weighted average is combined to evaluate the comprehensive condition of the studied object. According to the general principle of efficiency coefficient method, the evaluation score of single item index is as follows:

$$\;Y_{i}=\left(X_{is}-X_{min}\right)/\left(X_{max}-X_{min}\right)*C+D$$

In the formula, $X_{is}$ represents the variable original value, $X_{max}$ and $X_{min}$ respectively represent the maximum and minimum values of the variable; *C* and *D* are the known normal number, *C* is a multiple of ‘magnification’ or ‘reduction’ of the transformed value, *D* is the basis of the actual score. In most studies, generally take *C* = 40 and *D* = 60, thus, the base score is 60 and the highest score is 100.
